# Spatial Deconvolution of Cell Types and Cell States at Scale Utilizing TACIT

**DOI:** 10.21203/rs.3.rs-4536158/v1

**Published:** 2024-06-27

**Authors:** Khoa L. A. Huynh, Katarzyna M. Tyc, Bruno F. Matuck, Quinn T. Easter, Aditya Pratapa, Nikhil V. Kumar, Paola Pérez, Rachel J. Kulchar, Thomas J.F. Pranzatelli, Deiziane de Souza, Theresa M. Weaver, Xufeng Qu, Luiz Alberto Valente Soares Junior, Marisa Dolhnokoff, David E. Kleiner, Stephen M. Hewitt, Luiz Fernando Ferraz da Silva, Vanderson Geraldo Rocha, Blake M. Warner, Kevin M. Byrd, Jinze Liu

**Affiliations:** 1 Department of Biostatistics, Virginia Commonwealth University, Richmond, VA, USA.; 2 Massey Cancer Center, Richmond VA, USA.; 3 Lab of Oral & Craniofacial Innovation (LOCI), Department of Innovation & Technology Research, ADA Science & Research Institute, Gaithersburg, MD, USA.; 4 Department of Cell Biology, Duke University, Durham, NC, USA.; 5 Salivary Disorders Unit, National Institute of Dental and Craniofacial Research, National Institutes of Health, Bethesda, MD, USA.; 6 Adeno-Associated Virus Biology Section, National Institute of Dental and Craniofacial Research, National Institutes of Health, Bethesda, MD, USA.; 7 Department of Pathology, Medicine School of University of Sao Paulo, SP, BR.; 8 Division of Dentistry of Hospital das Clinicas of University of Sao Paulo, SP, BR.; 9 Laboratory of Pathology, Center for Cancer Research, National Cancer Institute, National Institutes of Health, Bethesda, MD, USA.; 10 Department of Hematology, Transfusion and Cell Therapy Service, University of Sao Paulo, Sao Paulo, Brazil.; 11 Division of Oral and Craniofacial Health Sciences, Adams School of Dentistry, University of North Carolina at Chapel Hill, Chapel Hill, NC, USA

**Keywords:** spatial biology, multimodal, transcriptomics, proteomics, artificial intelligence, machine learning, deep learning, multiplex imaging, fluorescence microscopy, brain, intestine, salivary gland, cell typing, single cell analysis, spatial multiomics

## Abstract

Identifying cell types and states remains a time-consuming, error-prone challenge for spatial biology. While deep learning is increasingly used, it is difficult to generalize due to variability at the level of cells, neighborhoods, and niches in health and disease. To address this, we developed TACIT, an unsupervised algorithm for cell annotation using predefined signatures that operates without training data. TACIT uses unbiased thresholding to distinguish positive cells from background, focusing on relevant markers to identify ambiguous cells in multiomic assays. Using five datasets (5,000,000-cells; 51-cell types) from three niches (brain, intestine, gland), TACIT outperformed existing unsupervised methods in accuracy and scalability. Integrating TACIT-identified cell types with a novel Shiny app revealed new phenotypes in two inflammatory gland diseases. Finally, using combined spatial transcriptomics and proteomics, we discovered under- and overrepresented immune cell types and states in regions of interest, suggesting multimodality is essential for translating spatial biology to clinical applications.

## INTRODUCTION

Spatial biology focuses on the precise understanding of the spatial distribution and relationship of cell types and their associated cell states within their native environments^1,2^. The field has been significantly advanced by rapidly expanding and maturing single-cell and spatial multiomics technologies, including established methods for transcriptomics and proteomics as well as emerging methods for spatial epigenomics, metabolomics, B cell and T cell receptor sequencing, and translatomics of open reading frames, each of which preserve the spatial context of cellular and architectural features, deepening our understanding of cellular interactions, biological pathways, and identifying new cell types that can be used as targets to improve disease treatments and precision diagnoses^3–8^.

The current era of spatial biology—characterized by single-cell and subcellular resolution, multi-omics technologies in nature and even combined modalities on a single tissue section—demands more advanced tools for scale and multimodality^9^. Among the multi-step bioinformatics workflow to support multiplex imaging data analyses^10,11^, identifying cell types and their cell states remains time-consuming and error-prone due to segmentation noise and signal bleed-through, restricted sets of molecular and protein panel markers, and multimodal marker-linked datasets^12^. The next innovations in the spatial biology field should address these issues in an assay-, species-, organ-, and disease-agnostic manner, considering scale and standardization.

Traditional unsupervised clustering methods commonly used in scRNA-seq analysis operate by grouping cells based on the overall similarity of their marker profiles across the entire panel^13–17^. Their efficacy heavily relies on the presence of abundant markers that distinguish cell populations, a characteristic commonly found in single cell sequencing data^18^. However, a significant challenge arises when dealing with predefined marker panels and cell types determined by as few as one marker^19^. This sparse marker set, often of only one modality, lacks power to separate expected cell populations in the embedded feature space, posing a formidable obstacle for unsupervised clustering to detect all cell types—especially rare ones^20^. Even with extensive parameter tuning combined with multi-step clustering to identify cell populations of interest, the desired results remain elusive^21,22^. Deep learning algorithms are increasingly utilized in spatial omics for cell type identification, but they require comprehensive and diverse training data to improve their accuracy and applicability to handle the complexities of spatial multiomics^23,24^.

**Figure 1.**
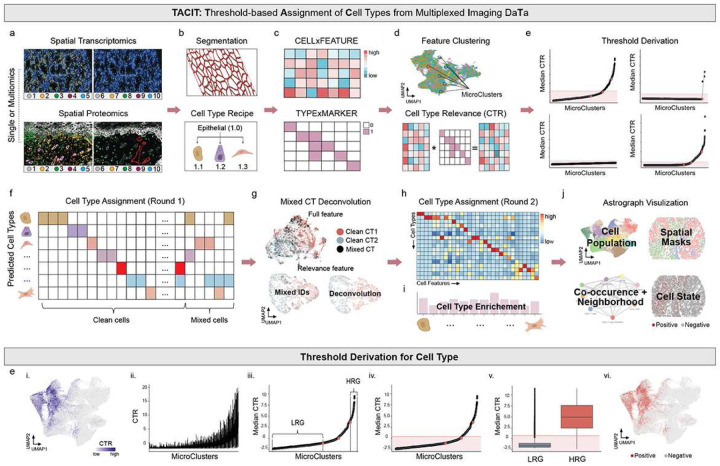
General TACIT Workflow: (a) Multiplex imaging employs both spatial proteomics (top) and spatial transcriptomics (bottom). After segmentation (b top), a CELLxFEATURE matrix is generated (c). Hierarchical cell type structures (b bottom) are formulated based on panel design, expert knowledge, and scRNA-seq marker matching, resulting in a CELLTYPExMARKER matrix (c). Cells are organized into microclusters (MCs) by a community-based Louvain algorithm, averaging 0.1%−0.5% of the population (d top). These matrices are then used to compute Cell Type Relevance (CTR) scores for all cell types across cells (d bottom). Optimal thresholds are established to classify cells as clean if they meet one threshold or mixed if multiple (e). The UMAP with all features shows no clear separation between two distinct cell types (g – top left); however, clear segregation appears when only relevant features are used in the UMAP embedding (g – top right). Mixed identities are resolved by analyzing the mode of cell types within their k-nearest neighbors (g – bottom). Validation is performed via heatmaps comparing mean marker and cell type values with the CELLTYPExMARKER matrix (h – top), and by calculating enrichment scores for each cell type (i – bottom). The UMAP plot illustrates spatial distributions with cell type annotations (j top-right) and connections of cell type clusters (j bottom-left), combining cell type and state analyses (j bottom-right). Extended details of step e: Threshold derivation extends to segmental regression on ordered median CTR scores across all MCs to identify breakpoints (i & ii), defining “low relevance group (LRG)” and “high relevance group (HRG)” (ii). The determined CTR threshold minimizes classification error, distinguishing between LRG and HRG (iv & v). Cells above the threshold are highlighted in red on the UMAP, while those below are in grey (vi).

To address these challenges, we developed **TACIT** (**T**hreshold-based **A**ssignment of **C**ell Types from Multiplexed **I**maging Da**T**a), an unsupervised algorithm for assigning cell identities based on cell-marker expression profiles. TACIT uses a multi-step machine learning approach to group cells into populations, maximizing the enrichment of pre-defined cell type-specific knowledge based from spatial transcriptomics and proteomics data (Fig. 1). Validated against expert annotation and available algorithms using five datasets from brain, intestine, and gland tissues in human and mouse, TACIT outperformed three existing unsupervised methods in accuracy and scalability. It also integrated cell types and states with a Shiny app to reveal new cellular associations in distinct immune-mediated exocrinopathies. Furthermore, we performed same-samples spatial transcriptomics and proteomics, finding that TACIT enabled RNA and protein agreement to 81% confidence. Thus, we demonstrate the inherent role for TACIT to support translational and clinical research applications through multimodal analyses.

## RESULTS

### Conceptualization of TACIT for Spatial Multiomics

TACIT is generally applicable to any probe-based, single-cell resolved spatial single modality or multimodal dataset (i.e., spatial transcriptomics or proteomics; Fig. 1a). Before TACIT can be employed, images containing tissues or cells are first segmented to identify cell boundaries (Fig. 1b). Features like probe intensity (protein antibodies) and count values (mRNA probes) are quantified, normalized, and stored in a single or multimodality CELLxFEATURE matrix (Fig. 1c). The TYPExMARKER matrix is derived from expert knowledge, with values between 0 and 1, indicating the relevance of markers for defining cell types (Fig. 1c).

TACIT conducts cell type annotation in two rounds. Cells are first clustered into microclusters (MCs) to capture highly homogenous cell communities with sizes averaging between 0.1–0.5% cells of the population using the Louvain algorithm (Fig 1d). In parallel, for each segmented cell, Cell Type Relevance scores (CTRs) against predefined cell types are calculated by the multiplication of normalized marker intensity vector with the cell type signature vector (Fig. 1d), quantitatively evaluating the congruence of cells’ molecular profile with considered cell types. The higher the CTR score, the stronger the evidence that the cell is associated with a given cell type. TACIT proceeds to learn a threshold that can separate cells into groups with strong positive signals and background noise (Fig. 1e). For a specific cell type, the median CTRs across all MCs are gathered (Fig. 1e^i^). The MCs are reordered by ranking its median CTRs values from lowest to highest (Fig 1e^ii^). The segmental regression model is fitted to divide the CTRs growth curve into 2 to 4 segments^25^. The two extremes of these segments represent the high relevance group and low relevance group, respectively. (Fig. 1e^iii^). A positivity threshold that minimizes the misclassification rates arising from cell outliers in both high relevance group and low relevance group is then established (Fig. 1e^iv^). Subsequently, the threshold is applied to all cells where the CTRs of cells exceeding the threshold for a specific cell type are labeled with positive, with the remaining labeled with negative (Fig 1e^v^–1e^vi^).

Cell labeling from the previous step can result in a single cell being labeled multiple cell types (Fig. 1f). To resolve the ambiguity, TACIT includes a deconvolution step (Fig. 1g) using the k-nearest neighbors (k-NN) algorithm on a feature subspace relevant to the mixed cell type category (Methods). The quality of cell type annotation is assessed by p-value and fold change, quantifying marker enrichment strength for each cell type (Fig. 1i) and visualized with a heatmap of marker expression (Fig. 1h). Following annotation, downstream analysis is performed using a custom, proprietary Shiny app we generated called **Astrograph** (Fig. 1j; Methods).

### Benchmarking TACIT Against Existing Unsupervised Algorithms

We downloaded two human datasets: Colorectal Cancer (PCF-CRC; n=140-TMAs; n=235,519-cells; n=56-antibodies) and Healthy Intestine (PCF-HI; n=64-samples; n=2,603,217-cells; n=56-antibodies); both were generated using the Akoya Phenocycler-Fusion (PCF; formerly CODEX) 1.0 system for spatial proteomics^26,27^. We compared TACIT’s performance in cell type annotation against CELESTA, SCINA, and Louvain in both datasets, using original annotations as reference^13,28,29^.

**Figure 2:**
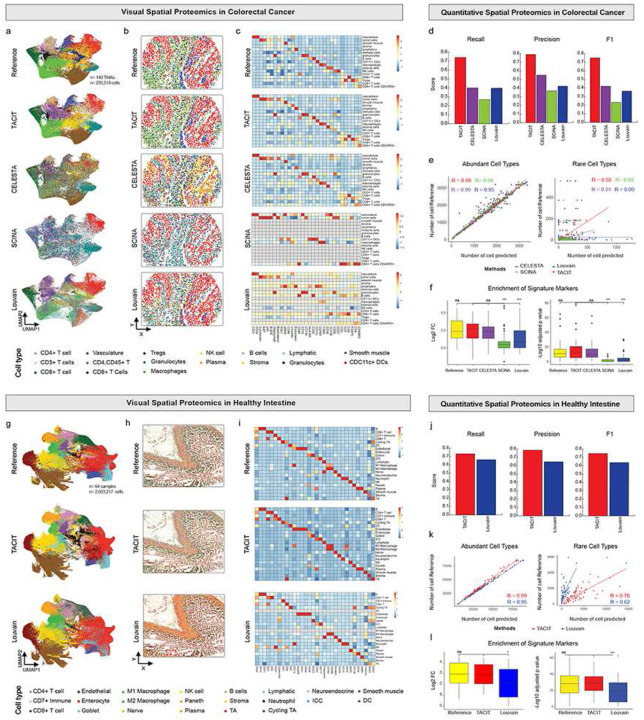
Application of TACIT on PhenoCycler data from PCF-CRC (top panel) and PCF-HI (bottom panel). (a,g) Examples of spatial plots color-coded by identified cell types, illustrating the spatial distribution and clustering of cells as determined by TACIT. These plots demonstrate how TACIT preserves the spatial structure of cell types, maintaining consistency with the reference data. (e,k) UMAP representations with cell type delineations, showing the clustering of cells in a two-dimensional space. TACIT’s UMAP plots reveal a higher degree of similarity to the reference clusters compared to other methods, indicating its superior performance in accurately identifying cell types. (f,i) Heatmaps comparing the mean marker values for each cell type identified by TACIT and other existing methods. TACIT’s heatmaps exhibit distinct and clear unique marker expressions for each cell type, with a diagonal pattern that highlights its precise cell type identification capabilities. (d,j) Recall, precision, and F1 score comparisons between TACIT (PCF-CRC: 0.74 (Recall), 0.79 (Precision), 0.75 (F1), PCF-HI: 0.73 (Recall), 0.79 (Precision), 0.75 (F1)) and existing methods, benchmarked against the reference. TACIT consistently outperforms other methods, achieving higher recall, precision, and F1 scores, which underscores its accuracy and reliability in cell type identification. (e,k) Correlation plots illustrating the relationships between different cell type identification methods for both abundant cell types and rare cell types. TACIT shows strong correlations with the reference data, particularly for rare cell types (PCF-CRC: R=0.58, PCF-HI: R=0.76), where it demonstrates a higher degree of similarity in cell type identification compared to other methods. (f,l) Intensity comparison of unique markers between TACIT and existing methods. TACIT displays significantly different enrichment scores, particularly when compared to methods like Louvain (PCF-CRC & PCF-HI: p-value<0.05) or SCINA (PCF-CRC: p-value<0.05), indicating its enhanced ability to identify and distinguish unique cell markers.

In the PCF-CRC dataset, TACIT demonstrated strong consistency with reference annotations compared and outperformed existing methods. This was evident through UMAP, spatial, and heatmap visualizations of cell populations, spatial patterning, and marker expression (Fig. 2a–c). As shown in the heatmap, Louvain failed to identify 6 out of 17 rare cell types, and SCINA identified only 5 total (Fig. 2c). TACIT achieved the highest accuracy, with weighted recall, precision, and F1 scores of 0.74, 0.79, and 0.75, respectively, significantly outperforming CELESTA, Louvain, and SCINA (p<0.05) (Fig. 2d; Extended Data 1). TACIT showed stable threshold and evaluation metrics in a bootstrap study (Extended Data 2a-d). For dominant cell types (≥1% of the population), TACIT, CELESTA, and SCINA exhibited high consistency (R=0.99) in terms cell type annotation, while Louvain slightly underperformed (R=0.95) (Fig. 2e). Both TACIT and CELESTA identified all expected rare cell types, though TACIT displayed a stronger correlation to the reference (R=0.58) compared to CELESTA (R=0.24) (Fig. 2e). Additionally, the accuracy for identifying rare cell types improved with an increasing number of resolutions (Extended Data 2e-f). Marker enrichment analysis indicated that TACIT annotations closely matched the signatures (Fig. 2f).

Derived from human intestine tissues, the PCF-HI dataset was used to test TACIT’s scalability and consistency. In this comparison, only Louvain was included, as CELESTA and SCINA labeled many cell types as “Others” (Extended Data 1). Both spatial and UMAP plots consistently showed TACIT aligning more closely with the reference (Figs. 2g, h). Louvain failed to detect dendritic cells and pro-inflammatory M1 macrophages, both present in the reference, highlighting TACIT’s accuracy in categorizing diverse cell types, especially clinically relevant ones like innate immune populations (Fig. 2i). TACIT outperformed Louvain with significantly higher recall (0.73 vs. 0.66), precision (0.79 vs. 0.64), and F1 scores (0.75 vs. 0.63) (Extended Data 1 and Fig. 2j). Both methods identified prevalent cell types with nearly perfect correlation (R=1.00), but TACIT excelled in identifying rare cell types, achieving a higher correlation (R=0.76) compared to Louvain (R=0.62; Fig. 2k). TACIT also showed significantly higher log2 fold change and -log10 p-adjusted values for unique cell type signatures than Louvain (p<0.0001) (Fig. 2l).

To evaluate TACIT’s performance on spatial transcriptomics data, we applied it to a published MERFISH dataset from the murine hypothalamic preoptic region of the brain (n=36-samples; n=1,027,848-cells; n=170-ISH panel)^30^. TACIT achieved significantly higher weighted recall (0.85), precision (0.87), and F1 scores (0.87) than Louvain (Extended Data 3a). Both methods showed high correlation with the reference for dominant cell types (R=0.99), but TACIT achieved higher correlation for rare cell types (R=0.94) compared to Louvain (R=0.64; Extended Data 3b). Spatial and UMAP plot demonstrated that cell type identification using TACIT closely matched the reference, with stronger and more distinct expression signatures than Louvain (Extended Data 3c-f). These findings underscore the effectiveness of TACIT in spatial transcriptomics. , providing reliable cell type identification for both abundant and rare populations.

### Applying TACIT to unpublished single modality spatial transcriptomics with linked scRNA-seq

**Figure 3:**
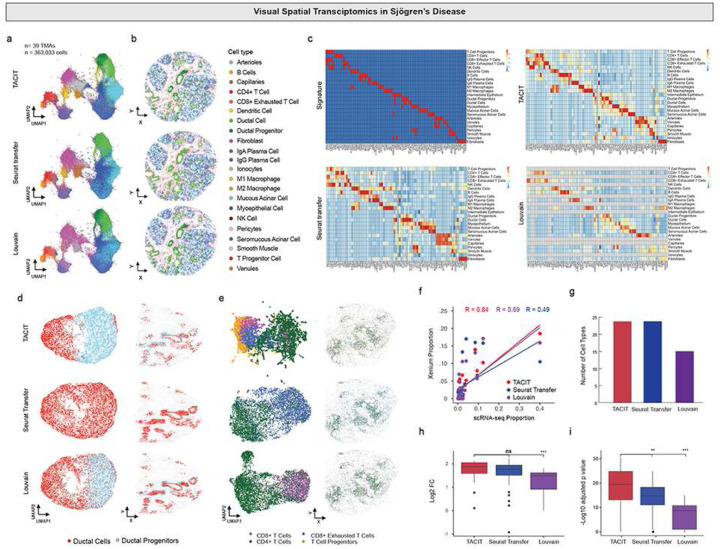
Application of TACIT on Xenium data. (a) UMAP and (b) spatial plots color-coded by identified cell types. The UMAP plots demonstrate TACIT’s ability to cluster cells accurately, showing a clear separation of different cell types. Epithelial such as mucous acinar, myoepithelial, and seromucous acinar cells form more distinct and clear clusters under TACIT’s annotation compared to Louvain and Seurat Transfer methods. The spatial plots further illustrate the spatial distribution of these cell types, maintaining the structural integrity and spatial organization consistent with the reference data. (c) Heatmaps depicting cell types and markers between TACIT, Louvain, Seurat transfer, and the signature matrix. TACIT’s heatmaps present clear and distinct patterns, highlighting its precise identification of cell types and markers. This clarity is especially notable when compared to the other methods, which show less distinct marker expressions. (d-e) UMAP plots with low granularity cell types across the three methods. TACIT’s enhanced capabilities are further exemplified by its identification of rare and diverse cell types, such as duct cells and duct progenitors, as well as various T cell types including CD4, CD8, CD8 exhausted, and T cell progenitors. (f) Correlation plot of cell type proportions between the three methods in Xenium, compared with scRNA cell type proportions. TACIT shows a higher correlation (Spearman Correlation, R=0.84) with scRNA cell type proportions, indicating a more consistent and reliable identification of cell types. In contrast, Seurat transfer and Louvain show lower correlations of 0.49 and 0.69, respectively. (g) TACIT and Seurat transfer able to find all the cell type matches with scRNA. (h-i) Intensity comparison of unique markers between TACIT and existing methods. TACIT exhibits a higher intensity of unique marker expressions compared to Louvain, with a log2 fold change (p-value<0.05), and shows significant performance over Louvain and Seurat transfer, with a -log10 adjusted p-value (p-value<0.05).

Next, TACIT was applied to an unpublished Xenium dataset (PI: Warner, NIH/NIDCR; n=21-patients; n=~360,000-cells; n=280-ISH panel) across 24 cell types. We compared TACIT against two annotation approaches: Seurat with label transfer from scRNA-seq data (Seurat transfer), and Louvain^29,31^. Signature lists for TACIT were created from the top five most enriched genes in each annotated cluster in the Seurat transfer result^32^. While the UMAP plot shows overall consistency in cell type annotation across the three methods, TACIT’s annotation excels in clear distinctions among three subtypes of acinar cells (Fig. 3a), corroborated by biologically meaningful spatial arrangement of these subtypes (Fig. 3b). TACIT demonstrated higher enrichment of signatures than both Louvain and Seurat transfer, with all cell types identified (Fig. 3c, h, I, g). Zooming into specific subtypes, TACIT clearly distinguishes ductal progenitors and ductal cells, while Seurat transfer labeled them all as “ductal cells” and Louvain showed mixed annotations (Figs 3d). TACIT also identified four subsets of T cells (CD4+, CD8+, CD8+ Exhausted, and Progenitors), which Louvain overlooked (Fig. 3e). This represents a critical populations to identify for autoimmune diseases i.e., Sjögren’s disease because T cell progenitors are crucial for maintaining immune tolerance, making them vital targets for therapeutic strategies and clinical applications in the future^33^. Overall, TACIT showed a strong correlation with scRNA-seq (R=0.84), higher than Seurat transfer (R=0.49) and Louvain (R=0.69) (Fig. 3f).

### Applying TACIT to unpublished same-slide spatial proteomics and transcriptomics

**Figure 4:**
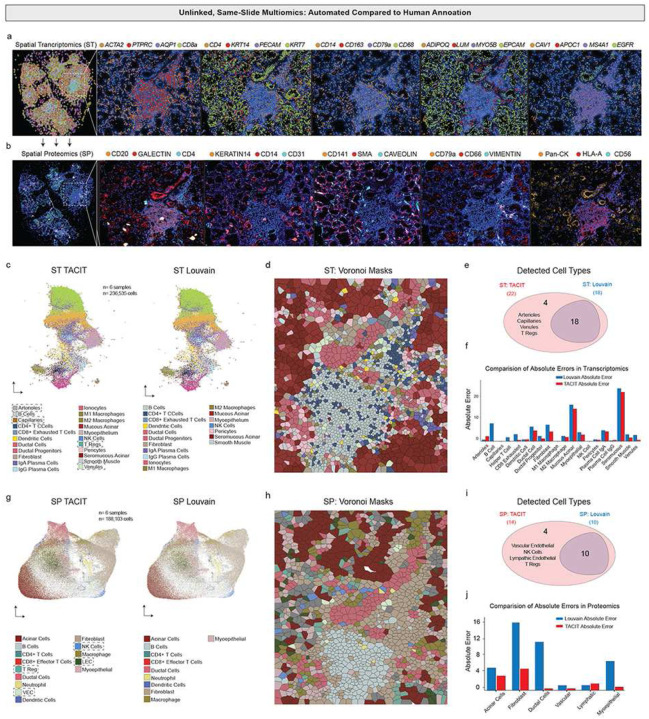
Single-Slide Spatial Multiomics Annotation using TACIT (a) A spatial transcriptomics experiment on minor salivary glands from GVHD patients used a Xenium platform with a 280-gene panel targeting structural and immune cells, revealing a high-density immune area with overlays of specific transcripts. (b) A subsequent spatial proteomics experiment on the same slide utilized a Phenocycler Fusion with a 36-antibody panel, sharing the segmentation mask for consistent spatial single-cell data extraction. (c) UMAP analysis of the Xenium data with TACIT and Louvain showed greater annotation granularity with TACIT, highlighting cell types identified only by TACIT (arrows). (d) A Voronoi plot for a GVHD case displayed detailed annotation reconstruction by TACIT, showing the heterogeneity in a dense immune infiltrate. (e) A Venn diagram demonstrated that TACIT identified 22 cell types, including four not matched by Louvain, although Louvain’s detected types were also identified by TACIT. (f) The absolute error in cell type assignments compared to human pathologist evaluations varied between TACIT and Louvain. (g) Another UMAP from the Phenocycler Fusion data emphasized TACIT’s higher granularity, with unique cell types marked (arrows). (h) A second Voronoi plot based on spatial proteomics data for a GVHD case illustrated TACIT’s annotation at a slightly lower resolution than the transcriptomics data. (i) A proteomics Venn diagram showed TACIT recognized and assigned 18 cell types, with two structural and two immune types uniquely detected. (j) The absolute error in cell quantity signatures from a spatial transcriptomics assay, compared with a human pathologist’s evaluation for each cell type, confirmed TACIT’s precision over Louvain.

To achieve detailed cell type annotation in spatial multiomics, we linked spatial proteomics (PI: Byrd, ADA Science & Research Institute; PCF 2.0; 36-antibody panel; Fig. 4a) and transcriptomics (Xenium; 280-ISH panel; Fig. 4b) on the same sample using segmentation mask transfer. This captured single-cell data for both TACIT and Louvain (see Methods; n=6-samples; 424,638-cells). Cellenics (now Trailmaker^®^) was used to generate cell type signatures (Extended Data 4). TACIT identified significantly more cell types than Louvain when applied to minor salivary glands affected by Graft-versus-Host Disease (GvHD), in both datasets (Extended Data 5,6; Figs. 4c–e). Louvain missed key cell types like vascular endothelial cells and TRegs. The reconstructed slide showed high immune cell density in the periductal region, indicating GvHD-associated immune infiltration (Fig. 4d). Compared to expert pathologist annotations, TACIT had a lower error rate than Louvain across all cell types (Figs. 4f).

In spatial proteomics, TACIT again identified 4 more cell types than Louvain (Figs. 4g,i), matching the spatial transcriptomic assignments and confirming GVHD-associated immune infiltration (Fig. 4h). TACIT uniquely identified vascular and lymphatic endothelial cells, Tregs, and NK cells (Fig. 4i). TACIT also had a lower mean error in annotating structural cell types, while Louvain over-assigned prevalent types like fibroblasts and ducts (Fig. 4j). The clinical impact of missing these cell types would prohibit precise understanding of MSG parenchymal changes and NK cell-mediated host cell death and IFN-γ and TNF-α release^34^,highlighting the importance of selecting the right tool for accurate cell annotation.

### Testing TACIT in linked spatial proteomics and transcriptomics ROIs

**Figure 5:**
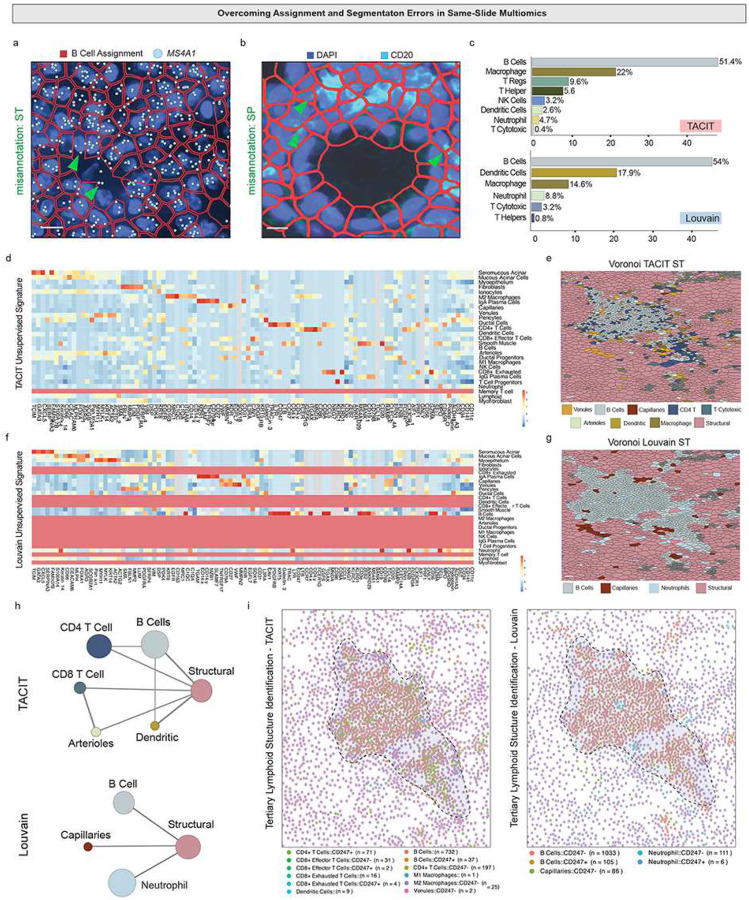
Application of TACIT in a Multimodal Single-Slide Tertiary Lymphoid Structure (a) Spatial transcriptomics and proteomics assays utilize segmentation to extract single-cell data, transferring the segmentation mask between experiments. However, this can lead to marker bleed-through; in proteomics, immunofluorescence markers stain the edges of adjacent B cells. In transcriptomics, probes such as the MS4A1 gene are found outside B cell boundaries in a GVHD minor salivary gland’s TLS. (b) TACIT and Louvain exhibit varying performances when analyzing high-density immune areas like a TLS. TACIT identifies a more detailed and expected population of immune cells within the TLS compared to Louvain. (c) A heatmap displays the genes and proteins used to create cell signatures by TACIT and Louvain. Despite using the same list of genes, TACIT outperforms Louvain by providing clearer markers for each cell type and more precise cell recognition in high-density immune areas. (d) Voronoi plots demonstrate how different cell assignments lead to varied analysis outcomes. TACIT’s reconstruction reveals a diverse mix of immune cells, small vessels, and antigen-presenting cells typical of a TLS. In contrast, Louvain shows lower resolution, merging all immune cells into broad categories of one innate and one adaptive type. (e) The choice of tools for cell assignment in multi-omics spatial assays impacts downstream analysis. The neighborhood analysis with TACIT illustrates expected cell proximities in a TLS, showing B cells and dendritic cells near small vessels and T cells. Conversely, Louvain shows unilateral interactions, focusing solely on the most abundant structural cell types in the analyzed ROI. (f) Using a single slide for spatial proteomics and transcriptomics allows for the identification of cell types and the assignment of specific biomolecules like chemokines, interleukins, and immune checkpoints to cells. This method not only reveals cellular patterns but also aids in studying spatial cell-cell communication. ROI reconstruction with TACIT assigned CD247 to T cells, B cells, and macrophages, highlighting diverse interactions. Conversely, the Clustering signature was exclusive to B cells, concentrated around capillaries, with no further interactions.

Because specific ROIs are often used for diagnosis or understanding disease pathophysiology, we decided to evaluate TACIT’s performance in confined areas. We selected nascent tertiary lymphoid structures (TLS) from GVHD for this application. TLSs pose unique challenges for spatial biology due to potential segmentation issues as they are highly concentrated with immune cells with large nuclei and little cytoplasm around diverse structural niches (epithelial, fibroblast, and vasculature)^35^. We applied a segmentation pipeline using a human-in-the-loop Cellpose3 model and still found areas in the TLS in both proteomic and transcriptomic space where signals like those for B cells (protein: CD20; mRNA: *MS4A1*) were misappropriated after segmentation Fig. 5a)^36^.

TACIT’s ability to deconvolve mixed cell phenotypes helps overcome segmentation errors. Within the TLS, TACIT identified more adaptive and innate immune cell types than Louvain, including TRegs and NK cells (Fig. 5b). Louvain detected fewer cell types with less distinct markers per cell type (Fig. 5c). In Voronoi reconstruction, Louvain identified TLS mainly composed of B cells, while TACIT showed primarily T cells surrounded by small vessels (Fig. 5d). Neighborhood analyses using Delaunay Triangulation and receptor-ligand pairs revealed different TLS phenotypes. TACIT showed expected relationships, such as proximity between dendritic cells and T cells, while Louvain showed structural-to-structural cell relationships (Fig. 5e). TACIT identified key markers for T cell exhaustion (PD-1/PD-L1 interactions) and small vessels essential for immune cell recruitment, while Louvain failed to detect vascular cells and showed less granularity in receptor-ligand assignments. This analysis demonstrates that niche- and disease-level phenotyping can be effectively captured using TACIT’s workflow.

### Multimodal Cell Identification with TACIT

**Figure 6.**
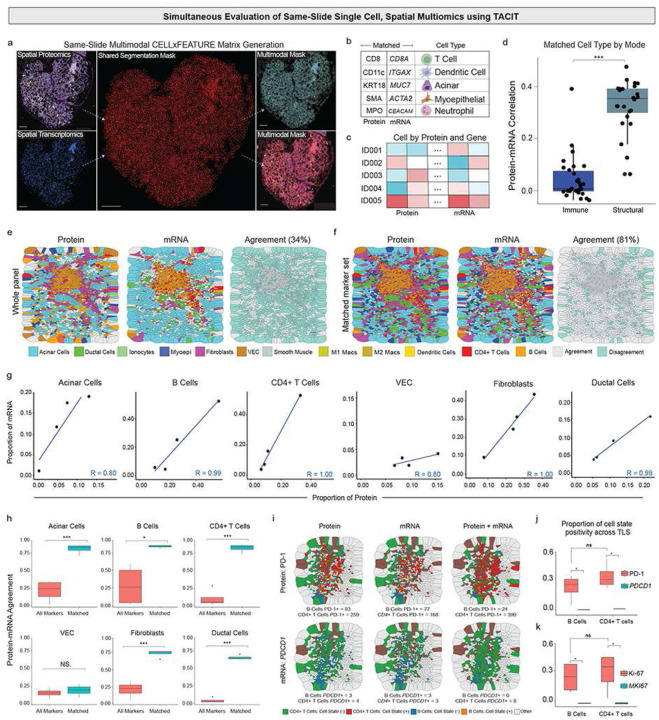
Multimodal analysis using ST and SP in a single slide. (a) Two assays were combined on the same slide and section: Phenocycler Fusion (SP) and Xenium (ST). A segmentation mask was created using a human-in-the-loop approach and inputted into the Xenium Ranger. This mask was then transferred to the SP assay, maintaining cell IDs between the two experiments.(b) After segmentation, a matrix was extracted containing the pixel values of each immunofluorescent channel from the SP and the transcripts per cell from the ST. (c) This cell-by-feature matrix was then normalized and cell-assigned using TACIT. (d). The matched number of cells assigned by the SP and ST assays was quantified to evaluate the correlation in cell assignment for each major cell type – structural and immune cells. The correlation for structural cells using all transcripts and proteins was 0.37, and for immune cells, it was 0.01. (e). After the initial annotation, specific cell markers were used to assign cell types that had both protein and transcript designations in the proteomics and transcriptomics assays. The masks of cells annotated in three different ROIs with a high density of immune cells showed 34% agreement when using all markers. (f). A smaller subset of matched protein and RNA panels was utilized to improve agreement. The Voronoi mask showed better convergence in cell type annotation, increasing cell ID matching to 81%. (g-h) The difference in annotation by each approach for each of the six cell types selected using matched protein and RNA markers showed an improvement in cell assignment, with the proportion of the cell types. (i). After multimodal cell assignment, TACIT was also able to provide cell state markers for each cell. PD-1 and *PDCD1* were used to understand the ratio of transcripts and proteins in high-density immune cell ROIs. The presence of these two markers was analyzed using SP alone, ST alone, and the two assays combined. (j) The proportion of positivity cell state in mRNA such as *PDCD1* and *MKI67* are significantly lower than PD-1 (p-value<0.05) and Ki67 (p-value<0.05) in protein for B cells and CD4+ T cells across TLS.

After collecting spatial transcriptomics (Xenium) and spatial proteomics (PCF) data, we used the same segmentation masks from Xenium on the PCF data, ensuring matched cell IDs for direct comparisons (see: Methods and Fig. 6a). This alignment allowed us to create a cell-by-protein and gene matrix for each cell, capturing both antibody intensities from PCF and count values from Xenium (Figs. 6b,c). Using TACIT, which incorporates marker signatures from both PCF and Xenium, we accurately identified cell types; other algorithms could not handle the multimodality for these assays. For the first time, the correlation of marker intensities between PCF and Xenium for immune cell markers was significantly lower than for structural cell types (p<0.0001) (Fig. 6d). Consequently, using the full marker panel on ROIs with many immune cells, the agreement between cell type identifications using only PCF markers versus only Xenium markers was about 34% (Fig. 6e and Extended Data 6a). However, focusing on markers common to both PCF and Xenium increased the agreement to 81% (Fig. 6f and Extended Data 7a). The proportion of cell types was high in the TLS between Xenium and PCF with higher agreement when using common markers (Figs. 6g,h). Importantly, for structural cell types like vascular endothelial cells (VEC) using our panel, they remained challenging to identify (see Fig. 4).

For effective clinical translation, it is crucial to accurately assign both spatial cell identity and state. To address this, we tested *PDCD1*/PD-1, a key component of the immune checkpoint inhibitor (ICI) pathway. Comparing the same markers across both technologies revealed differences in cell states, particularly between PD-1 and *PDCD1* across all four TLS (Fig. 6i and Extended Data 7b). These results were statistically significant for B cells and CD4+ T Cells (Fig 6.j). The same trend followed for cell cycling marker Ki-67/*MKI67* (Fig. 6k). This is clinically relevant because accurately predicting the cell cycle and PD-1 expression in B cells and CD4+ T cells is crucial for optimizing immunotherapy, as it helps identify which patients will benefit most from treatments like checkpoint inhibitors^37^. The differences observed across all three recipes—unimodal and multimodal—highlight the importance of understanding which factors are truly critical for patient outcomes, especially considering they varied with spatial scales in cell and sample number.

## DISCUSSION

Identifying cell types in multiplex imaging-based spatial omics data is a significant challenge with current technologies. Unsupervised clustering methods like Louvain produce inconsistent results based on resolution, UMAP dimensionality, or programming language (R vs. Python)^38^. Finding optimal parameters is time-consuming and often suboptimal^24^. In contrast, TACIT automates cell type annotation, mimicking manual gating with enhanced scalability and precision. This method excels in phenotyping based on multiplex panel design, effectively identifying both dominant and rare cell populations without bias. The success of TACIT stems from its initial focus on cell type-specific features—beginning with the evaluation of cell type-specific markers and followed by the deconvolution of mixed cells within relevant subspaces. This approach is crucial for identifying cell types in spatial transcriptomics and proteomics, where specific features can be sparse (transcriptomics), or less markers (proteomics). Additionally, the TACIT method is exceptionally scalable, designed to efficiently handle datasets containing up to 2 million cells on a standard laptop with 16GB of memory in a single run. This capability is critical for processing extensive spatial transcriptomics data, allowing researchers to perform comprehensive analyses without requiring specialized high-performance computing resources.

Benchmarking TACIT on three public spatial omics datasets, which include nearly 5 million cells across 51 cell types, demonstrates its broad applicability as a tool agnostic to assay, species, organ, and disease for precise cell type annotation. Additionally, TACIT’s application to the Xenium dataset, initially annotated by scRNA-seq data through label transfer, further showcases its effectiveness in refining cell type annotations and discovering cell type specific markers through exploratory analysis.

The combined analysis of spatial multiomics datasets in GVHD revealed the importance of integrating spatial transcriptomics and proteomics for deep phenotyping. PCF and Xenium data differ in that PCF provides continuous values while Xenium provides count data, and there is often a lack of correlation between corresponding markers, especially immune ones^39^. The discrepancies in our final dataset, which combines transcriptomics and proteomics on a single slide, highlight the need for better-designed multimodal panels to accurately identify cell types in spatial datasets. This data prompts a reevaluation of how we define both known and yet-to-be-discovered cell types in complex biological systems. Looking forward, cell type identification is poised for a significant transformation through the integration of advanced omics technologies—such as transcriptomics, proteomics, spatial epigenomics, metabolomics, B cell and T cell receptor sequencing, and translatomics—alongside traditional histological stains like H&E, PAS, Masson’s trichrome, and Picrosirius Red-polarization. These advancements will enhance the depth of analysis possible with limited marker sets, offering high-resolution insights into gene and protein expression that enable precise cell classification beyond traditional approaches and current spatial biology techniques.

TACIT has its limitations. TACIT relies upon accurate cell type signatures to achieve robust cell type classification. Additionally, TACIT requires sufficient sampling of cells with abundant background signals to derive relevant thresholds, making it less appropriate when applied to small regions with few cells. Additionally, TACIT may leave some cells unassigned due to poor marker intensities, inaccurate segmentation, or the presence of undefined cell types. An in-depth analysis of unassigned cells may reveal novel cell types that are not characterized by existing cell type signatures.

By providing detailed cell type annotations and uncovering rare cell populations, tools like TACIT can eventually enhance the accuracy of prognostic assessments and treatment planning. Additionally, the technology’s ability to analyze spatial multiomics data enables the identification of unique cellular neighborhoods and their interactions, which is critical for understanding disease progression and therapeutic response in the near future as part of clinical research and ultimately, precision clinical care. As TACIT continues to evolve, its application in personalized medicine could lead to the development of tailored treatment regimens based on the specific cellular composition and state of individual patients’ tissues, improving outcomes and reducing adverse effects. Furthermore, ongoing advancements in automation and machine learning could enhance TACIT’s scalability and robustness, making it an indispensable tool in clinical research and precision immunology, rheumatology, oncology, and beyond.

## METHODS

### CELLxFEATURE matrix

Let *M* be a set of markers used in a spatial omics panel, |M|=m, and *N* be the set of cells of size *n* captured in a tissue slide. Let *A*_*n*×*m*_ be the CELL by FEATURE information captured in the spatial omics experiment following cell segmentation process. For spatial proteomics such as PhenoCycler, entry *a*_*ij*_ in the matrix A represents the z-normalized intensity value indicating the Intensity level of a specific marker *j* within cell *i*. In the context of spatial transcriptomics such as Xenium or MERFISH/MERSCOPE, *a*_*ij*_ reflects the log-normalized of the count of transcripts for each gene.

### Cell type signature matrix

Let T be a set of cell types, |T| = t, to be captured by the panel. We define a cell signature matrix *S*_*m×t*_ of markers that define individual cell types, where each element *s*_*ij*_ in *S*

$$s_{ij}=\{\begin{array}{cc} w,\;0<w\leq 1, & \text{if}\;\text{marker}\;i\;\text{serves}\;\text{as}\;a\;\text{singnature}\;\text{of}\;\text{cell}\;\text{type}\;j\\ & 0,\;\text{otherwise} \end{array}$$

The value *w* indicates the importance of a specific marker in defining a cell type. If such information is not available, *w* is set to 1 by default.

### Cell type relevance matrix

Let Γ denote a cell type relevance matrix, with dimension *n* × *p*, where *n* is the number of profiled cells, and *p* is the number of cell types included in the panel. The cell type relevance (CTR) score is computed using the formula:

Γ=A*S

where each element in Γ provides a quantitative measure of a cell’s relevance to a specific cell type. By summing up the relevant markers’ intensity values weighted by their importance (set to 1 by default), we can directly measure a cell’s marker intensity profile alignment with the expected cell type signature. For each cell type, a cell with higher CTR score suggests a stronger association between the observed marker intensities with the expected signature of a specific cell type, indicating a higher likelihood that the cell belongs to that cell type.

### Micro-clustering

Louvain clustering method from the Seurat version 5 toolkit was applied on the CELLxFEATURE matrix *A* to conduct the fine-grained clustering of cells^31^. The resolution of the clustering was set so that the average number of cells per cluster remained between 0.1% to 0.5% cells of the entire population. We refer to the resulting clusters as a collection of microclusters (MCs) denoted as Φ = {*c*_1_, *c*_2_, …, *c*_*k*_}. These microclusters are expected to be highly homogeneous, capturing a group of cells with highly similar marker profiles and thus with high likelihood to represent cells of the same cell type. The distribution of marker values across all markers in Φ will be used to approximate the variations of marker values across the diverse cell populations they represent.

### Segmented regression model

Next, to identify MCs with distinct cell type relevance, we employed segmented regression model aiming to identify specific breakpoints at which the relationship between the MCs changes^25^. For any given cell type, the median CTR scores across all *k* MCs are calculated and stored as a vector *z* = (*z*_1_, *z*_2_, … *z*_*k*_) = (*r*_1_, *r*_2_, …, *r*_*k*_) be a vector where *r*_*i*_ is the rank of *z*_*i*_ in *z*. Next, a segmental regression model is fitted with *z* being the dependent variable and *r* as the predictor to identify breakpoints that divide the data into distinct linear segments.


$$z=\alpha_{0}+\beta_{0}r+\sum_{i=1}^{g}\beta_{i}{\left(r-\varphi_{i}\right)}_{+}$$

Where:

- α_0_ represents the intercept of the linear model,
- *β*_0_ represents the slope of the linear segment before the first breakpoint,
- *β*_*i*_ represents changes in slope at the breakpoint *i*,
- *g* represents number of breakpoints,
- φ_i_ represents the optimal location of breakpoint *i*, and
- (*r* − φ_i_), is defined as max(0, *r* − φ_i_) for breakpoint *i*.

Our proposed method aimed to obtain an optimal fitting by allowing a maximum of three breakpoints. This was determined by the minimal Akaike Information Criterion (AIC) score achieved among the three models the three models (g=1, 2 and 3)^40^. The breakpoints from the optimal model were then utilized to categorize clusters into either “low” or “high” relevance groups, Φ_L_ and Φ_H_, respectively. Specifically, the MCs ranking below the lowest breakpoint were classified as $\Phi_{\text{L}}=\{i|r_{i}\leq \;\varphi^{1},1\leq i\leq \kappa \}$, where *r* is the vector containing the rank positions of MCs. Correspondingly, the MCs ranking above the highest breakpoint were considered as high relevance group $\Phi_{\text{H}}=\{i|r_{i}\geq \;\varphi^{\text{max}(g)},\;1\leq i\leq \kappa \}$.

### Optimal threshold

Next, an optimal CTR threshold to differentiate positive and negative cells of a given cell type was determined as follows. Let *C*_*L*_ denote the set of cells that belong to MCs within Φ_L_, formally defined as $C_{L}=\cup_{i\;\in \;\Phi_{\text{L}}}c_{i,}c_{i}\in \Phi$. Similarly, *C*_*H*_ is the set of cells that belong to MCs within Φ_H_, defined as $C_{H}=\cup_{i\;\in \;\Phi_{\text{H}}}c_{i,}c_{i}\in \Phi$. Each MC encompasses a range of CTR scores, suggesting that even within a highly homogeneous cluster, there is relatively broad range of marker intensity. The preferred threshold minimizes the misclassification rate between the two relevance groups. This optimization problem aims to find a threshold (*θ*) that minimizes the number of cells in the low relevance group *C*_*L*_ with CTR scores exceeding the threshold, and the number of cells in the high relevance group *C*_*H*_ with CTR scores lower than the threshold. The grid search with this objective function can be expressed with the formula:

- $\theta =\text{argmin}(|\{i|\tau_{i}\;>\;\theta ,i\;\in \;C_{L}\}\left|+\right|\{i|\tau_{i}<\theta ,i\;\in \;C_{H}\}|)$, where:
- θ represents a desired optimal threshold for a given cell type,
- τ_i_ is the CTR score for cell *i*,

### Cell Type Categorization

After determining an optimal threshold of CTR score for each cell type, cells exceeding this threshold were marked as positive, while the rest were marked as negative. Applying this threshold to each cell type resulted in a binary matrix B of dimension *n* × *p*, with 1 indicating a cell is positive or 0 indicating negative. Based on the positivity of individual cells across cell types, cells were categorized into three distinct sets:

1. Clean cells: The set of cells classified as positive for exactly one cell type.
2. Mixed cells: The set of cells classified as positive in more than one cell type, suggesting a blend of characteristics from multiple cell types.
3. Unknown cells: The set of cells that are not classified as positive for any cell type.

### Deconvolution of Mixed Cells

The set of mixed cells underwent a process of cell type deconvolution to assign each cell to its final cell type. This step leveraged two outcomes from the previous step. Firstly, a significant portion of cells classified as clean cells in each individual cell type may now serve as anchor cells to resolve the cells with mixed identities. Secondly, even though more than one identity is assigned as candidates for mixed cells, a vast majority of cell types were recognized as irrelevant and were eliminated from further consideration. Thus, the classification algorithm focused on the relevant markers and excluded irrelevant markers.

Let *ξ*, *ξ* ⊂ T, be a combination of cell types deemed positive in a set of cells, denotes as $N_{\xi }^{\text{mix}}$. Additionally, all the clean cells positive in each of cell types in *ξ* were also extracted, denoted as $N_{\xi }^{\text{clean}}$. Let *M*_ξ_ be the set of markers serving as signatures for cell types in *ξ*. Next, a submatrix from matrix *A*, denotes as *A*_*ξ*_, containing the intensity values of both the clean cells and the mixed cells, i.e., $N_{\xi }=N_{\xi }^{\text{clean}}\;\cup \;N_{\xi }^{\text{mix}}$, in the marker set *M*_*ξ*_ was extracted. The *k*-nearest neighbors (KNN) algorithm is applied to cell feature matrix *A*_*ξ*_ to classify the cells with mixed identities in *ξ*^41^. For each mixed cell in $N_{\xi }^{\text{mix}}$, the algorithm works by first calculating its relative distances to clean cells within *ξ*-relevant markers in *M*_*ξ*_. This step is crucial as it utilizes only the signature markers for *ξ*, eliminating noise and biases from irrelevant markers in the deconvolution of cell types in *ξ*. The *k* neighbors that are closest to each of the mixed cells were identified according to their distance. Finally, the identity of a cell was determined by the mode of the identities of its *k*-nearest clean cell neighbors (*k* =10 by default).

### Comparisons with other methods

We compared our proposed method with three existing cell phenotyping methods, namely CELESTA, SCINA, Louvain + manual annotation clusters, and Seurat transfer using scRNA. The code for CELESTA, SCINA, Louvain annotation and Seurat v5 transfers label scRNA methods are publicly available for reproducibility and comparison purposes.

### CELESTA^28^

CELESTA is a cell type identification algorithm for spatial proteomics that uses an optimization framework to assign individual cells to their most likely cell types based on prior knowledge of each cell type’s marker signatures. It utilizes a marker-scoring function to match a cell’s marker expression probability profile to known cell type signatures. In our application, CELESTA was run for each of the tissue microarrays (TMAs). The major function included CreateCelestaObject() to create celesta object. FilterCells() to filter out cells that are artifact, with high_marker_threshold = 0.9, and low_marker_threshold=0.4. AssignCells() function to assigned cell types, with max_iteration=10, and cell_change_threshold=0.01. For each cell type, Additional parameters including high_expression_threshold_anchor, low_expression_threshold_anchor, high_expression_threshold_index, and low_expression_threshold_index needed to be defined. For consistency, the default setting was used as provided in this GitHub (https://github.com/plevritis-lab/CELESTA/tree/main). For PCF-HI datasets, CELESTA labeled all cells as Unknown even with the high_expression_threshold_anchor levels set at 0.2.

### SCINA^13^

SCINA is a method used for cell type identification in scRNA-seq, employing a combination of cell type-specific marker signatures and an expression matrix. Data normalization is performed through log-transformation before further annotation. A signature matrix (referenced in Table S1) is utilized to classify cell types. In the first phase, primary cell types such as vasculature, tumor cells, stroma, immune cells, and smooth muscle are identified. Cells labeled as immune or unknown in the first round undergo a second round of classification, where they are further distinguished into B cells, T cells, CD11c+ dendritic cells, natural killer cells, lymphatics, plasma cells, macrophages, and granulocytes. The third round focuses on cells categorized as T cells or unknown from the second round, aiming to specify subsets like CD4 T cells, CD8 T cells, regulatory T cells (Tregs), and CD45RO+ CD4 T cells. For the PCF-HI, most of the cells returned Unknown, which could not be included in the analysis. The SCINA algorithm is executed using the SCINA() function, with parameters such as max_iter = 100, convergence_n = 10, convergence_rate = 0.999, sensitivity_cutoff = 0.9, rm_overlap=TRUE, allow_unknown=TRUE, and log_file=‘SCINA.log’. For more information about SCINA, refer to https://github.com/jcao89757/SCINA.

#### Louvain^29^:

Louvain clustering is a widely used unsupervised method for identifying cell types in spatial omics datasets. This technique, originally developed for community detection in networks, optimizes modularity to partition data into clusters, making it particularly effective for distinguishing distinct cell populations based on gene expression profiles. To run Louvain clustering on spatial omics data, we first normalized the data using z-score normalization to standardize the expression levels. Next, we scaled the data to ensure that each feature contributed equally to the analysis. We then performed dimensionality reduction using Uniform Manifold Approximation and Projection (UMAP) on the first 30 principal components to visualize the data in a lower-dimensional space. Finally, we applied Louvain clustering on the UMAP dimensions with a resolution of 0.8 to identify distinct clusters. After that, FindMarkers() function in Seurat version 5 was used to find the top 5 markers that define the clusters^31^. We looked at individual clusters with their expression to assign cell types and the top 5 markers to assign the cell type for each cluster.

#### Seurat Label Transfer^31^:

Automatic cell labeling was informed by the scRNAseq dataset using post-quality control data. Subsequent data scaling was performed using the ScaleData() function. Dimension reduction was achieved through PCA and UMAP, utilizing the RunPCA() and RunUMAP() functions respectively, focusing on the 30 selected features. The method involved the FindTransferAnchors function from Seurat v5. All 25 clusters remained consistent between the reference (SC) and query (ST) objects.

### Performance metrics

#### Compare with reference:

For a specific cell type, True Positive (TP) calls are defined as cells where the assigned cell types from the method match those in the ground truth benchmark dataset. False Positive (FP) calls are cells where the assigned cell types by the method do not match the ground truth or reference. False Negative (FN) calls represent cells assigned by the benchmark but not by the method, while True Negative (TN) calls are cells not assigned by either the method or the benchmark. The weighted score considers the proportion of each cell type in the reference dataset, where *i* is a cell type in the set of reference.


$$\text{Accuracy}=\frac{TP+TN}{TP+FP+TN+FN}$$

$$\text{Weighted}\;\text{recall}=\sum_{i}\left(\frac{TP_{i}}{TP_{i}+FN_{i}}\right)*\text{Proportio}n_{i}$$

$$\text{Weighted}\;\text{precision}=\sum_{i}\left(\frac{TP_{i}}{TP_{i}+FP_{i}}\right)*\text{Proportio}n_{i}$$

$$\text{Weighted}\;\text{F}1=2*\frac{\text{Weighted}\;\text{precision}*\text{weighted}\;\text{recall}}{\text{weighted}\;\text{precision}+\text{weighted}\;\text{recall}}$$

### Benchmark Datasets

Four multiplexed tissue imaging studies with high confidence cell type assignments were used for TACIT evaluation and benchmarking:

#### PhenoCycler 1 (PCF-Colorectal cancer)^26^:

Data representing 140 tissue microarray (TMA) spots from 35 colorectal cancer (CRC) patients (17 in the CLR group and 18 in the DII group) were collected from 36 distinct tissues. In this study, the authors used spatial proteomics to examine the tumor environment and how the immune response correlates with survival outcomes in colorectal cancer. The TMAs were collected and imaged using a 56-marker CODEX (CO-Detection by indEXing) panel, profiling a total of 258,386 cells. Cells identified as immune/vasculature (n=2,153) and immune/tumor (n=1,797), along with cells lacking a marker signature—including adipocytes (n=1,811), nerves (n=659), undefined (n=6,524), monocytes (n=815), and cells categorized as dirt (n=7,357)—were excluded from the analysis. This exclusion resulted in 235,519 cells being retained for the cell type annotation benchmark analysis. The TMA imaging was segmented based on DRAQ5 nuclear stain, pixel intensities were quantified, and spatial fluorescence compensation was performed using the CODEX toolkit segmenter (available at https://github.com/nolanlab/CODEX). Subsequently, the cells were subjected to X-shift clustering, and the resulting clusters were manually annotated to ensure the accuracy of the cell labels. The list of signature was provided in the original paper^26^. PCF-CRC can be download at: https://data.mendeley.com/datasets/mpjzbtfgfr/1.

#### PhenoCycler 2 (PCF-Human Intestine)^27^:

Data from 64 sections of the human intestine were collected from 8 donors (B004, B005, B006, B008, B009, B010, B011, and B012). In this study, the authors used spatial proteomics to examine the structure of the large and small intestines in humans. The raw image data were segmented using either the CODEX Segmenter or the CellVisionSegmenter (available at https://github.com/nolanlab/CellVisionSegmenter). Employing a 57-marker CODEX panel, a total of 2,603,217 cells were profiled. These cells were initially grouped using Leiden clustering and subsequently annotated under the supervision of the authors^42^. To ensure accuracy, the cell type labels were further consolidated by the authors by inspecting back-annotated cell types on the original images. The list of signatures cell types was provided in the original paper and expert domain knowledge. PCF-HI can be download at: https://datadryad.org/stash/dataset/doi:10.5061/dryad.pk0p2ngrf.

#### MERFISH^30^:

The mouse brain datasets included data for 36 mouse sample IDs across a total of 60 slides. In this study, by combining MERFISH with scRNA-seq, we elucidated the molecular, spatial, and functional organization of neurons within the hypothalamic preoptic region. The raw image data were segmented using a seeded watershed algorithm with DAPI and total mRNA co-stains. Initially, 1,027,848 cells were profiled. These cells were classified using graph-based clustering and subsequently annotated by the authors. For our analyses with TACIT, we excluded 153,080 cells labeled as ‘Ambiguous.’ Additionally, to comply with Louvain’s method requirements, cells where over 70% of genes had zero counts were also removed. The list of signatures cell types was provided in the original paper. After these filtering steps, the dataset prepared for comparison with Louvain includes 505,961 cells covering 170 genes. MERFISH can be downloaded at: https://datadryad.org/stash/dataset/doi:10.5061/dryad.pk0p2ngrf.

#### Xenium-SjD:

A tissue microarray (TMA) was constructed, consisting of 63 cores derived from formalin-fixed paraffin-embedded (FFPE) tissue blocks from 21 patients (11 with Sjögren’s Disease (SjD) and 10 without). Three cores per tissue block were extracted, using a TMA array to organize the blocks, and the patient samples were randomized from 1 to 21. To fit within the fiduciary framework of the TMA, the section was divided in half by scoring, placing 44 cores on a single slide, including 8 additional cores designated for control tissues. The analysis utilized the standard 280-plex Human breast cancer panel according to the protocols provided by 10x Genomics.

#### Xenium-GVHD:

A tissue microarray including three patients with chronic graft-versus-host disease and three healthy minor salivary glands, derived from FFPE tissue blocks, was mounted on a Xenium Slide (10x Genomics). To fit within the fiduciary frame, we melted the original blocks and embedded the samples in one block. The analysis utilized the standard 280-plex human breast cancer panel from 10x Genomics according to the protocol provided by the company.

### Marker Enrichment Strength

For each marker unique to a specific cell type (a marker that is a signature for only one cell type), we calculated the log2 fold change (log2FC) of that marker in the signature cell type compared to the mean value in other cell types where it is not a signature. Additionally, we performed a one-sided Wilcoxon test to determine if the expression of the marker in the signature cell type was significantly greater than its expression in non-signature cell types.

### Statistical Analyses

Statistical analyses were conducted, and figures were created using R (version 4.3.0). For comparisons between two groups, Student’s t-test was used when the assumption of normality was met; otherwise, the non-parametric Wilcoxon rank-sum test was applied. For comparisons involving more than two groups, analysis of variance (ANOVA) was used, followed by post-hoc tests if significant differences were detected. For multiple comparisons, the false discovery rate was used to adjust the P-values (Benjamini-Hochberg procedure). Results were considered statistically significant if P < 0.05 or if the adjusted P < 0.05 for multiple testing.

### Cell-cell interactions and neighborhood analysis

Spatial omics data from each individual tissue was processed that describes cellular interactions as graphs with nodes representing individual cells and edges potential cellular interactions as determined by Delaunay triangulation. A 97^th^ percentile distance threshold was established for each tissue to eliminate edges representing improbably long cell-to-cell distances. Cells classified as “Unknown” (non-deconvoluted cells) were excluded from the analysis before conducting Delaunay triangulation. An interaction matrix was then constructed, with each element *a*_*ij*_ representing the number of edges shared between cell type *i* and cell type *j*. To visually represent these differences, a hierarchically clustered heatmap using Euclidean distance was generated.

### Shiny app

The Shiny app (here called **Astrograph**) takes the input of the signature matrix and the CSV file output from TACIT annotation, which includes spatial information, UMAP coordinates, CELLxFEATURE matrix, and marker thresholds. The app provides a user interface with spatial plots and UMAP visuals featuring annotations, marker expression thresholds, and weighted cell type calculations. Users can also access color annotations, spatial neighborhood connections between cell types across the whole tissue or ROI, and Moran’s I for each marker and cell type to identify spatial autocorrelation. Additional tools include annotated mean heatmaps, Voronoi plots, and proportions of cell types and cell state markers.

### Trailmaker^®^

The single-cell RNA sequencing dataset was managed, analyzed, and visualized using the Trailmaker^®^ community platform (https://scp.biomage.net/) hosted by Biomage (https://biomage.net/). Cellenics is now Trailmaker^®^, just released by Parse Biosciences. Pre-filtered count matrices were uploaded to Trailmaker^®^. Barcodes were filtered through four sequential steps. Barcodes with fewer than 500 UMIs were removed. Barcodes representing dead or dying cells were excluded by filtering out those with more than 15% mitochondrial reads. A robust linear model was fitted to the relationship between the number of genes with at least one count and the number of UMIs per barcode using the MASS package (v. 7.3–56) to filter outliers. The model predicted the expected number of genes for each barcode, with a tolerance of 1 - alpha, where alpha is 1 divided by the number of droplets in each sample. Droplets outside the prediction interval were removed. The scDblFinder R package v. 1.11.3 was used to calculate the likelihood of droplets containing multiple cells, and barcodes with a doublet score above 0.5 were filtered out. After filtering, each sample contained between 300 and 8000 high-quality barcodes, which were then input into the integration pipeline. Initially, data was log-normalized, and the top 2000 highly variable genes were selected using the variance stabilizing transformation (VST) method. Principal component analysis (PCA) was performed, and the top 40 principal components, explaining 95.65% of the total variance, were used for batch correction with the Harmony R package. Clustering was performed using Seurat’s implementation of the Louvain method. For visualization, a Uniform Manifold Approximation and Projection (UMAP) embedding was calculated using Seurat’s wrapper for the UMAP package. Cluster-specific marker genes were identified by comparing cells of each cluster to all other cells using the presto package’s Wilcoxon rank-sum test. Keratinocytes were isolated from the complete experiment by extracting manually annotated barcodes and filtering the Seurat object. These subset samples were then input into the Biomage-hosted instance of Trailmaker^®^. Filtering steps were skipped since the data was already filtered. The data underwent the same integration pipeline as the full experiment. All cells were manually annotated using relevant literature and CellTypist.

### Ethical Approval

All original research (Figures 3–5; Extended Data 5) complies with country-specific regulations for ethical research engagement with human participants.

Sample Collection and Tissue Preparation: Deidentified minor salivary gland (MSG) tissues were obtained from diagnostic biopsies in healthy and chronic GVHD patients (University of Sao Paulo IRB 65309722.9.0000.0068; MTA 45276721.4.0000.0068 IRB/MTA). All patients seen at the Dentistry Division of the Hospital das Clinicas of Medicine School of University of Sao Paulo reported herein provided informed consent before participation in this research protocol. All patients have received full medical and dental assistance during the research time and will be followed by the oral medicine team unrestricted. Tissues were fixed in a 10% solution of NBF for a minimum of 24 h at 4°C and mounted on paraffin-embedded SuperFrost Plus slides (See Methods for biopsy and tissue-mounting procedures).

NIH research participants were seen in the NIDCR Sjögren’s Disease Clinic and provided informed consent to NIH Single IRB-approved protocols (15-D-0051, NCT00001390) before any study procedures were performed. All participants were assessed and categorized based on the 2016 classification criteria from the American College of Rheumatology (ACR) and the European League Against Rheumatism (EULAR)^43^. Comparator tissues were obtained from subjects (non-SjD) who were otherwise healthy and did not meet the 2016 ACR-EULAR criteria. Participants underwent standardized screening for systemic autoimmunity and received thorough oral, salivary, rheumatological, and ophthalmological evaluations. Clinical investigations adhered to the principles outlined in the Declaration of Helsinki.

#### Clinical Protocol University of Sao Paulo:

Patients included in this study were sourced from two distinct pathways. One pathway involved direct inclusion from the São Paulo Capital Death Verification System. This included patients who had died from acute causes and were under 65 years of age. These individuals underwent post-mortem minor salivary gland biopsies within 4 hours of death. Tissue removal was performed using the minimally invasive autopsy technique^44^. The collected tissue samples were then sent to the histology department at the University of São Paulo School of Medicine for further processing as outlined in the described protocol.

GVHD patient biopsies were obtained from the biobank at the University of São Paulo School of Medicine. These patients were re-consented and followed up for chronic GVHD clinical evaluation. The biopsy samples, taken during episodes of oral lesions, were sent to the histology department for processing following the same procedures mentioned above.

#### Spatial Transcriptomics (Xenium) Sample Preparation:

The Xenium workflow was performed according to manufacturer protocols (10x Genomics 5 μm FFPE tissue sections were sectioned onto a Xenium slide,deparaffinized, and permeabilized to make the mRNA accessible. A 313-probe mRNA panel, containingand two negative controls to assess non-specific binding and genomic DNA (gDNA) controls, was used in this study.. Probe hybridization occurred overnight at 50 °C with a probe concentration of 10 nM. After a stringent wash to remove un-hybridized probes, the probes were ligated at 37 °C for 2 h, during which a rolling circle amplification (RCA) primer annealed. The circularized probes were then enzymatically amplified (1 h at 4 °C followed by 2 h at 37 °C), which produced multiple copies of the gene-specific barcode for each RNA binding event andresulted in a high signal-to-noise ratio. After washing, background fluorescence was chemically quenched. Sections were then placed into an imaging cassette for loading onto the Xenium Analyzer instrument.: We used the 280-gene Xenium Human Breast Panel for healthy and GvHD MSG analyses. Once the experiment was finished, slides were stored in a 50% glycerol solution.

#### Xenium Analyzer Instrument:

The Xenium Analyzer is a fully automated system that includes an imager (with an imageable area of approximately 12 × 24 mm per slide), sample handling, liquid handling, wide-field epifluorescence imaging, capacity for two slides per run, and an on-instrument analysis pipeline. The imager uses a fast area scan camera with a high numerical aperture, a low read noise sensor, and approximately 200 nm per-pixel resolution. Image acquisition on the Xenium Analyzer is performed in cycles. The instrument automatically cycles in fluorescently labeled probes for detecting RNA, incubates, images, and removes them. This process is repeated for 15 rounds of fluorescent probe hybridization, imaging, and probe removal, with Z-stacks taken at a 0.75 μm step size across the entire tissue thickness.

#### Image Pre-Processing:

The Xenium Analyzer captures Z-stacks of images in every cycle and channel, which are then processed and stitched to create a spatial map of the transcripts across the tissue section. Stitching is performed on the DAPI image, taking all stacks from different fields of view (FOVs) and colors to create a complete 3D morphology image (morphology.ome.tif) for each stained region. Lens distortion is corrected based on instrument calibration data, which characterizes the optical system. The Z-stacks are further subsampled to a 3 μm step size, which is empirically determined to be useful for cell segmentation quality. Image features are extracted from overlapping FOVs and feature matching estimates offsets between adjoining FOVs to ensure consistent global alignment across the image. Finally, the 3D DAPI image volumes (Z-stacks) generated across FOVs are stitched together.

#### Spatial Proteomics (Phenocycler Fusion):

The spatial proteomics GvHD was performed on 5 μm FFPE sections mounted on SuperFrost Plus slides (ThermoFisher, MA, USA). The sections underwent deparaffinization and rehydration, followed by immersion in a Coplin jar containing 1:20 AR9 buffer (Akoya Biosciences, MA, USA). The jar was placed in a pressure cooker for 15 min at low pressure, then cooled at room temperature for 30 min. After rinsing in deionized water and 100% ethanol, the slides were immersed in hydration buffer for 2 min and staining buffer for 20 min (Akoya Biosciences, MA, USA). The primary antibody cocktail was prepared according to the manufacturer’s protocol (see Table 1 for a complete list of antibodies)^35^. The slides were then placed in a humidity chamber (StainStray, Sigma-Aldrich, MO, USA) and, *in a change to the manufacturer protocol*, incubated overnight at 4°C. Following incubation, slides were fixed in a post-staining solution for 10 min. After fixation, slides underwent sequential 1-minute PBS washes and a 5-min immersion in ice-cold methanol. The sections were then treated with 200 μL of a final fixative solution for 20 minutes, followed by additional washes to remove the fixative. Slides were dried and mounted using the Akoya flow cell device, which sealed the flow cell onto the slides, for 30 s. The slides were removed from the press and soaked in 1X PCF buffer (Akoya Biosciences, MA, USA). PCF reporter stock solution was prepared according to manufacturer instructions and was distributed into 18 amber vials, with each vial containing 235 μL of the solution. For each cycle, 5 μL of reporter was added to each vial, resulting in a total volume of either 245 μL (for 2 reporters) or 250 μL (for 3 reporters) as detailed in Supplemental Methods Table 2. Reporters were selected from Atto550, AlexaFluor 647, and AlexaFluor 750 based on experimental needs. Distinct pipette tips were used to transfer the contents of each amber vial into a 96-well plate. DAPI-containing vials were pipetted into wells in the H-row, while reporter-containing vials were distributed into other rows. Once the wells were filled, they were sealed with adhesive aluminum foil (Akoya Biosciences, MA, USA). Imaging was conducted using a PhenoCycler Fusion 2.0 with a 20X objective lens (Olympus). Solutions required for instrument operation included nuclease-free water, 1X PCF buffer with buffer additive, and low- (20%) and high-concentration (80%) DMSO in 1X PCF buffer, prepared by adding appropriate volumes of DMSO to 1X PCF buffer with additive using a stir plate.

Antibody List and Reporter List

**Table:** 

PCF Antibody	Clone	Barcode/Reporter	Wavelength
CD8A	C8/144B	BX/RX026	Atto550
CD4	EPR6855	BX/RX003	AF647
CD20	L26	BX/RX020	AF750
GZMB	D6E9W	BX/RX041	Atto550
FOXP3	236A/E7	BX/RX031	AF647
Ki67	B56	BX/RX047	Atto550
PHH3	AKYP0060	BX/RX030	AF647
HLA-A	EP1395Y	BX/RX004	AF750
Galectin-3	M3/38	BX/RX035	Atto550
CD3E	EP449E	BX/RX045	AF647
CD45RO	UCHL1	BX/RX017	Atto550
CD45	D9M81	BX/RX021	AF647
CD21	AKYP0061	BX/RX032	Atto550
PD-L1	73–10	BX/RX043	AF647
CD14	EPR3653	BX/RX037	Atto550
PD-1	D4W2J	BX/RX046	AF647
MPO	AKYP0113	BX/RX098	Atto550
CD68	KP1	BX/RX015	AF647
CD31	EP3095	BX/RX001	AF750
KRT14	Poly19053	BX/RX002	Atto550
CD107a	H4A3	BX/RX006	AF647
KRT8/18	C51	BX/RX081	AF750
CD141	AKYP0124	BX/RX087	Atto550
ICOS	D1K2T	BX/RX054	AF647
SMA	AKYP0081	BX/RX013	AF750
PDPN	NC-08	BX/RX023	Atto550
COL_IV	EPR20966	BX/RX042	AF647
CD34	AKYP0088	BX/RX025	Atto550
HLA-DR	EPR3692	BX/RX033	AF647
Bcl2	EPR17509	BX/RX085	AF647
Caveolin	D46G3	BX/RX086	AF750
IFNG	AKYP0074	BX/RX020	Atto550
CD66A/C/E	ASL-32	BX/RX016	AF647
CD56	CAL53	BX/RX028	Atto550
CD11c	118/A5	BX/RX024	AF647
PanCK	AE-1/AE-3	BX/RX019	AF750

#### Image Segmentation:

qpTIFF images were opened into QuPath 5.0,. The segmentation was acquired in three different methods. The linear nuclei expansion was obtained using Watershed directly from QuPath. The Pre trained models were used applying the QuPath extension generated using the workflow established by Bankhead P. (https://qupath.readthedocs.io/en/latest/docs/advanced/stardist.html).

The HITL methods utilized used a GUI based approach established by Cellpose 3.0 with denoising and HITL training in 50 different ROIs of MSG H&E sections. The application of the methods was performed into a 3 ROIs of 900 microns x 800 microns in three different GVHD patients MSG biopsies. The parameters used by the three methods were the same: Pixel size was 0.1 μm, Sigma 1, DAPI threshold 12. Cell expansion was 10 into the linear model and the pre-trained model, and no cell expansion was required for the HITL model. In the HITL model the mask was exported to QuPath, allowing the same extraction .csv matrix with the cell IDs and the protein markers expressed in each cell ID.

#### Protocol. Combined Xenium and PCF:

After the Xenium experiment, the slides underwent a quenching process as described in the Xenium Assay 10X Genomics manual. The slides were then stored in a container with 50% BPS and 50% glycerol for two days. To resume the experiment, the slide was washed in PBS for 3 minutes, and antigen retrieval was performed using AR9 Buffer (Akoya Biosciences) in a pressure cooker for 15 minutes at low pressure. The rest of the antigen retrieval protocol until the start of the PhenoCycler fusion experiment was carried out as described in the ‘spatial proteomics’ methods section above.

#### Mask Transfer:

For the combined Xenium and PCF assay, the cell segmentation masks obtained from Xenium analyzer were used for both Xenium and PCF analysis. Since Xenium acquisition is performed with a 40x objective lens and PCF with a 20x objective lens, for the purposes of cell mask transfer (from Xenium to PCF), the Xenium DAPI image (morpholopgy_mip.ome.tif) was down sampled by a factor of 2. The Xenium cell boundary polygons (stored in cell_boundaries.csv.gz in the Xenium output folder) were subsequently converted to match the downsampled Xenium DAPI image. The cell boundary masks were then saved as a .geojson, with their cell names from Xenium analyzer retained, for use in QuPath for subsequent analysis. Since it is possible for the sample not be perfectly aligned Xenium and PCF experiments, the PCF .qptiff image was registered to the down sampled Xenium DAPI image, using the non-rigid registration workflow in VALIS v1.0.4 (https://www.nature.com/articles/s41467-023-40218-9). The resulting aligned PCF image was saved as an .ome.tiff with the additional downsampled Xenium DAPI channel using the Kheops plugin for FIJI (Guiet, R., Burri, O., Chiaruttini, N., Seitz, A., & Eglinger, J. (2021). Kheops (Version 0.1.8) [Computer software]. https://doi.org/10.5281/zenodo.5256256).

#### Manual Quantification:

For the comparison of cell assignment methods, manual counting was conducted by a pathologist (BFM) within designated Regions of Interest (ROIs). These ROIs comprised 1500–1800 cells each. Manual counting involved quantifying cells based on canonical marker labels and morphological features. For example, KRT18 combined with specific morphological features was used to identify Acinar Cells, PAN-Ck combined with morphological features identified Duct cells, CD31 identified Vascular endothelial cells, SMA identified Myoepithelial cells, and CD45 identified immune cells. Additionally, specific markers were utilized for identifying unique cell types that are determined by a single marker. Upon completion of the manual counting process, the quantification data were systematically transferred into a table format. This table facilitated the calculation of the presence of each cell type within the respective ROIs. To assess the convergence between clusters and TACIT, the average number of cells for each type was used to compute the absolute error associated with each cell type.

## Data Availability

The benchmark public data (PCF-CRC, PCF-HI, and MERFISH) can be found at: https://data.mendeley.com/datasets/mpjzbtfgfr/1 (PCF-CRC), https://datadryad.org/stash/dataset/doi:10.5061/dryad.pk0p2ngrf (PCF-HI), and https://datadryad.org/stash/dataset/doi:10.5061/dryad.pk0p2ngrf (MERFISH). Source data for the reproduced figures, pair PCF and Xenium (cell by proteins/genes) are available at: https://zenodo.org/records/11397609 . All other data is available upon reasonable request.
